# Breast metastasis of small bowel carcinoid tumor misdiagnosed as primary breast cancer

**DOI:** 10.4103/0256-4947.55317

**Published:** 2009

**Authors:** Armin Shahrokni, Mohammad R. Rajebi, Muhammad W. Saif

**Affiliations:** From the Yale University School of Medicine, New Haven, CT, USA

**To the Editor:** Carcinoid tumors are rare, but constitute the most common gastrointestinal neuroendocrine tumors.^1^ These tumors arise from enterochromaffin cells of the gastrointestinal tract. Carcinoid syndrome occurs in approximately 50% of patients with intestinal carcinoids and manifests as episodes of diarrhea, abdominal pain, and flushing.^2^ Small intestinal carcinoids metastasizing to the breast have only sporadically been reported in the literature.^3^ Metastatic breast carcinoid can be easily mistaken for primary breast carcinoma. This may potentially be detrimental for the patient, especially if the primary surgery is a mastectomy with axillary lymph node dissection.

A 64-year-old Caucasian female presented to her primary care physician with a chief complaint of left nipple pain. Mammogram showed a 2-cm solid mass in the lateral aspect of left breast suspicious for malignancy. Ultrasound-guided biopsy showed papillary carcinoma. Subsequently, a staging CT scan showed multiple hypodense lesions throughout the liver consistent with metastatic disease. Biopsy of the largest lesion confirmed the metastatic nature of the disease.

Diagnosis of stage IV papillary carcinoma of the breast was made and the patient was started on bevacizumab and paclitaxel. Following her initial treatment, the patient remained in good performance status except that she continued to complain of worsening diarrhea and facial flushing, which she had had for the last seven years prior to the current disease and which was attributed to menopause.

**Figure 1:**
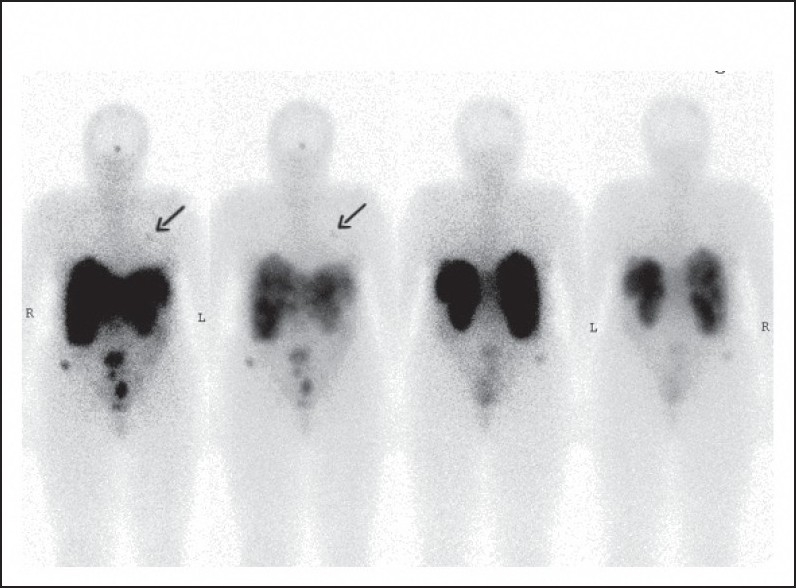
In-DTPA pentetreotide (octreotide) scan of the patient showing multiple areas of abnormal radiotracer activity within the abdomen, left breast (arrow), and calivarium.

The persistent symptoms led the patient to seek a second opinion at the Yale Cancer Center. An octreotide scan showed multiple areas of increased activity within the liver, abnormal accumulation in the lower abdomen, upper pelvis, left midthorax and mid frontal and left superior parietal region of the calvarium (Figure 1).

**Figure 2:**
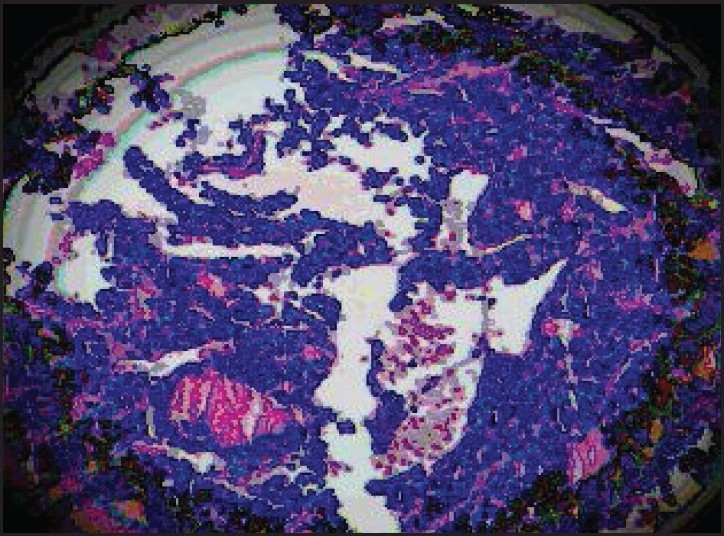
Breast biopsy of the patient showing a large nest of carcinoid tumor, infiltrating normal parenchyma (hematoxylin and eosin, ×40).

**Figure 3:**
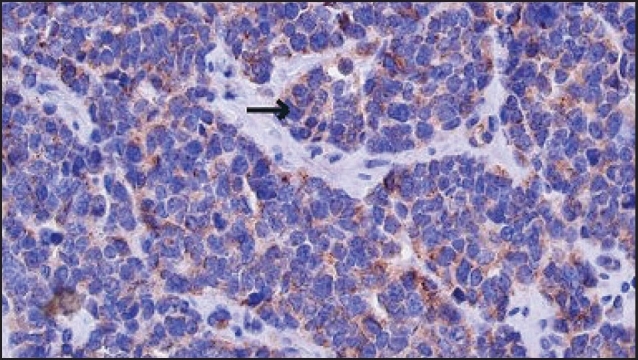
Breast biopsy of the patient showing immunoreactivity for chromogranin A.

Laboratory data showed increased pancreatic polypeptide (347 pg/mL; normal, up to 80 pg/mL) and serotonin (1448 ng/mL; normal, 101-283 ng/mL) levels. A previous breast biopsy was reviewed by the pathologist revealed a well-differentiated infiltrating neuroendocrine carcinoma (NET). Synaptophysin and chromogranin were intensely and diffusely positive confirming neuroendocrine differentiation (Figures 2, 3). Based on these findings, a diagnosis of NET of the small bowel was made with metastases to the liver and left breast. Lumpectomy of the breast was performed and chemotherapy was stopped. The patient has been treated with octreotide injection. Two years later, the patient is active and radiological imaging shows stable disease with no new areas of metastases.

Carcinoid tumors in the breast are rare. These tumors may occur as metastases from a known carcinoid tumor, or as a primary presentation of a metastatic carcinoid tumor, or as a primary breast tumor (Table 1).^4^ Carcinoid tumors contain characteristic cytoplasmic granules that can be highlighted with special stains such as chromogranin, synaptophysin, or neuron-specific enolase.^5^ Patients with carcinoid tumor metastatic to the breast should undergo lumpectomy alone, with multiple resections if more than one lesion is present. Patients with primary breast carcinoid should be treated in a manner similar to invasive ductal breast cancer that is appropriate for the size and stage of the lesion.^6^ It is important to differentiate between primary breast carcinoma, primary breast carcinoid tumor and metastatic disease to the breast because of differences in treatment.

**Table 1:** Available data of reported cases with primary and metastatic carcinoid tumor to the breast.

	Average age, yr (range)	Unilateral/bilateral	Location of primary	Misdiagnose as breast cancer before surgery	Carcinoid syndrome
History of carcinoid tumor (n=5)	54 (20-72)	11/1	Ileocecal, appendix, duodenum, pancreas, lung	6/15	4/15
Breast as first presentation (n=9)	56 (35-97)	8/1	Ileocecal, appendix, ovary	6/9	6/9
Primary breast carcinoid (n=38)	66 (35-97)	26/3	Breast	8/38	0/38
